# Designing a stepped wedge trial: three main designs, carry-over effects and randomisation approaches

**DOI:** 10.1186/s13063-015-0842-7

**Published:** 2015-08-17

**Authors:** Andrew J. Copas, James J. Lewis, Jennifer A. Thompson, Calum Davey, Gianluca Baio, James R. Hargreaves

**Affiliations:** MRC London Hub for Trials Methodology Research, MRC Clinical Trials Unit at University College London, Aviation House, 125 Kingsway, London, WC2B 6NH UK; MRC Tropical Epidemiology Group, Department of Infectious Disease Epidemiology, London School of Hygiene and Tropical Medicine, Keppel St, London WC1E 7HT, UK; Department of Infectious Disease Epidemiology, London School of Hygiene and Tropical Medicine, Keppel St, London WC1E 7HT, UK; Department of Social and Environmental Health Research, London School of Hygiene and Tropical Medicine, Keppel St, London WC1E 7HT, UK; Department of Statistical Science, University College London, Gower Street, London, WC1E 6BT, UK

**Keywords:** Stepped wedge trials, Design, Methodology, Public health

## Abstract

**Background:**

There is limited guidance on the design of stepped wedge cluster randomised trials. Current methodological literature focuses mainly on trials with cross-sectional data collection at discrete times, yet many recent stepped wedge trials do not follow this design. In this article, we present a typology to characterise the full range of stepped wedge designs, and offer guidance on several other design aspects.

**Methods:**

We developed a framework to define and report the key characteristics of a stepped wedge trial, including cluster allocation and individual participation. We also considered the relative strengths and weaknesses of trials according to this framework. We classified recently published stepped wedge trials using this framework and identified illustrative case studies. We identified key design choices and developed guidance for each.

**Results:**

We identified three main stepped wedge designs: those with a closed cohort, an open cohort, and a continuous recruitment short exposure design. In the first two designs, many individuals experience both control and intervention conditions. In the final design, individuals are recruited in continuous time as they become eligible and experience either the control or intervention condition, but not both, and then provide an outcome measurement at follow-up. While most stepped wedge trials use simple randomisation, stratification and restricted randomisation are often feasible and may be useful. Some recent studies collect outcome information from individuals exposed a long time before or after the rollout period, but this contributes little to the primary analysis. Incomplete designs should be considered when the intervention cannot be implemented quickly. Carry-over effects can arise in stepped wedge trials with closed and open cohorts.

**Conclusions:**

Stepped wedge trial designs should be reported more clearly. Researchers should consider the use of stratified and/or restricted randomisation. Trials should generally not commit resources to collect outcome data from individuals exposed a long time before or after the rollout period. Though substantial carry-over effects are uncommon in stepped wedge trials, researchers should consider their possibility before conducting a trial with closed or open cohorts.

## Background

Stepped wedge cluster randomised trials (SWTs) are becoming increasing popular and are being applied to a growing range of interventions, as shown in our review article [1]. However, SWTs encompass a broad range of designs, and the methodological literature is lagging behind the growth in the conduct of SWTs. Much of the literature to date has focussed on a small range of SWT designs where data are collected from individuals at discrete time points, and individuals contribute one measurement during the study [2–5]. This may, for example, arise from cross-sectional sampling from all clusters just before each crossover point (whenever a group of clusters changes from control to intervention condition). However, most SWTs described in the recent literature do not follow this particular design [1]. Consequently, there is limited published guidance for planning SWTs, and adapting the published guidance to the broad range of designs in use is not straightforward.

Researchers planning SWTs must consider a range of design issues, starting with how individuals from within clusters will participate. The design literature makes little distinction between SWTs where individuals are exposed to one condition only, or to both control and intervention conditions. The literature has also not clearly addressed the role of data collected before and/or after the rollout period in the study. The limited range of designs considered has also hampered the growth of terminology to describe the conduct of SWTs, and allow them to be reported in a transparent and consistent way, though others have begun this process [6].

In this paper we formally define the characteristics of SWTs, including aspects of the cluster allocation strategy for an SWT, and describe the range of ways in which individuals might participate in terms of exposure and measurements. We describe which key aspects should be reported and the role of graphical presentation. We review recent SWTs to identify the most commonly conducted designs and illustrate each with a case study. We describe which designs we think provide high quality evidence and those where the potential for bias, principally from carry-over effects, should be carefully considered and investigated. Cluster definition, individual exposure, and participation are largely determined by the research question and setting. We describe and guide the key design choices in planning an SWT: the randomisation method, the number of steps and length of time between successive crossover points, whether the trial will be complete or incomplete, and whether data shall be collected before or after the rollout period. Sample size is the topic of another article in this series [7].

## Methods

We developed a framework and terminology by which to define the key characteristics of an SWT and the cluster allocation. We then developed a typology of individual exposure and measurement, and with that in mind, examined the recently published SWTs reviewed in this series [1] to identify commonly used designs and illustrative case studies. We considered the strengths and weaknesses of the commonly used designs, focussing on the possibility of carry-over effects. We identified the key design choices for an SWT and developed guidance for each one. We also considered how the design of an SWT can be clearly reported. The work presented here did not require ethical approval as it involves only critical thinking and review of published research articles.

## Results

### Defining characteristics of stepped wedge cluster randomised trials, allocation and terminology

An SWT is a trial in which clusters receive the intervention at different time points, the order in which they receive it is randomised, and data are collected from clusters over time.

**Fig. 1:**
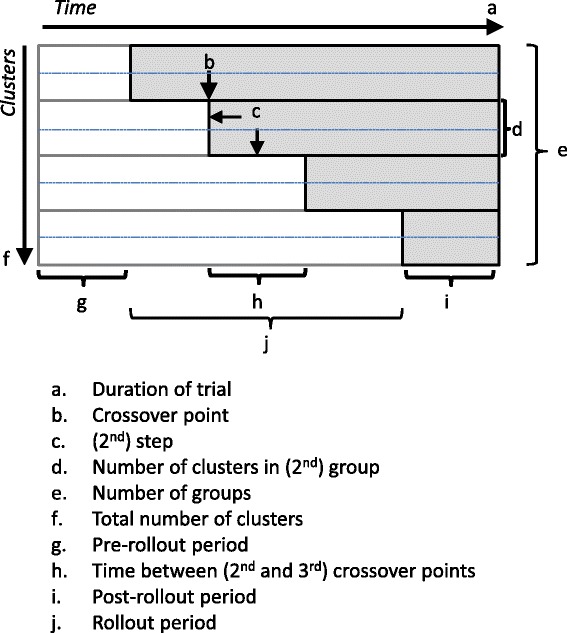
Characteristics and terminology of stepped-wedge cluster randomised controlled trials, where shaded areas indicate intervention exposure and unshaded areas indicate control exposure

Figure 1 identifies the key features that define the allocation strategy for an SWT. SWTs randomly allocate clusters to groups that cross over from a control condition to an intervention at different crossover points (b). Key aspects of the allocation strategy are the number of clusters per group (d), the number of groups (e), and the length of time between successive crossover points, sometimes referred to informally as the ‘step length’ (h), which together also determine the total number of clusters (f) and total trial duration (a). In Figure 1 there are four groups, each with two clusters. We define a step in the design to be both a crossover point and the time to the subsequent crossover point (c).

SWTs can have up to three main phases. For all SWTs data will be collected during a rollout period (j), in which groups of clusters are crossing over from the control condition (often standard care or policy) to the intervention condition. At any one time during this period, some clusters are allocated to the intervention condition while others are not. In SWTs there may also be periods of data collection before the rollout period (g) and/or after the rollout period (i). In some trials, individuals are exposed to the control and/or intervention condition within the trial, but are then measured later after a (potentially long) follow-up period. In such trials we consider outcome data from individuals exposed before, during, or after rollout to be ‘collected’ before, during, or after rollout.

### Typology of individual exposure to intervention and control conditions and measurement

Like other cluster randomised trials (CRTs), SWTs are generally designed to study the effects of a new intervention, such as a policy or staff training programme which is implemented at the cluster level, but experienced and measured by its impact on individuals. For example, in a situation where hospital staff implement a new patient management policy, the SWT is designed to establish whether this leads to better outcomes for patients who are treated at the hospital.

Whilst the clusters in an SWT normally participate throughout the trial, experiencing control and intervention conditions at different times according to the allocation strategy, the ways in which individuals are exposed and participate vary greatly between trials. For example, in some SWTs, all individuals participate in the trial from start to end and experience both control and intervention conditions. In other SWTs, all individuals who participate experience either the control or intervention for only a brief exposure period (for example, a hospital appointment), and the outcome may be measured after a follow-up period, that is, a period in which individuals are no longer exposed to the control or intervention condition but are still required to participate in order to measure the effect of the intervention. These features of how individuals participate normally reflect how such individuals experience treatments and/or policies in the wider population outside the trial, and how they become eligible and cease being eligible in the population, for example how they first attend hospital and how they are discharged from care. These features are often outside the control of the trialists, but influence how SWTs are designed. In some SWTs, all participating individuals contribute one or more outcome measurements. In other SWTs where large clusters (such as cities) are randomised, then only a small fraction of the participants may be invited to provide outcome measurements, for example by a questionnaire survey.

In order for SWT designs to be fully reported and to enable readers to judge their strengths and weaknesses, it is important to describe how individuals participate in a trial, how they are exposed to control and/or intervention conditions, and how measurements are obtained. In a recent article, Hemming *et al*. described three SWT designs, but these mainly considered how measurements are obtained from individuals, and not features of participation or exposure [8]. We first describe three common designs for SWTs identified in our review of 37 trials, then briefly outline characteristics by which the individual participation exposure and measurement can be identified and reported in an SWT. In the supplementary table of our review [1], each SWT is assigned to one of these three designs, aside from two trials following non-standard designs described later. For each design we offer one detailed example from the review.

### Three main stepped wedge cluster randomised trial designs: individual exposure and measurement

#### Continuous recruitment with short exposure

**Fig. 2:**
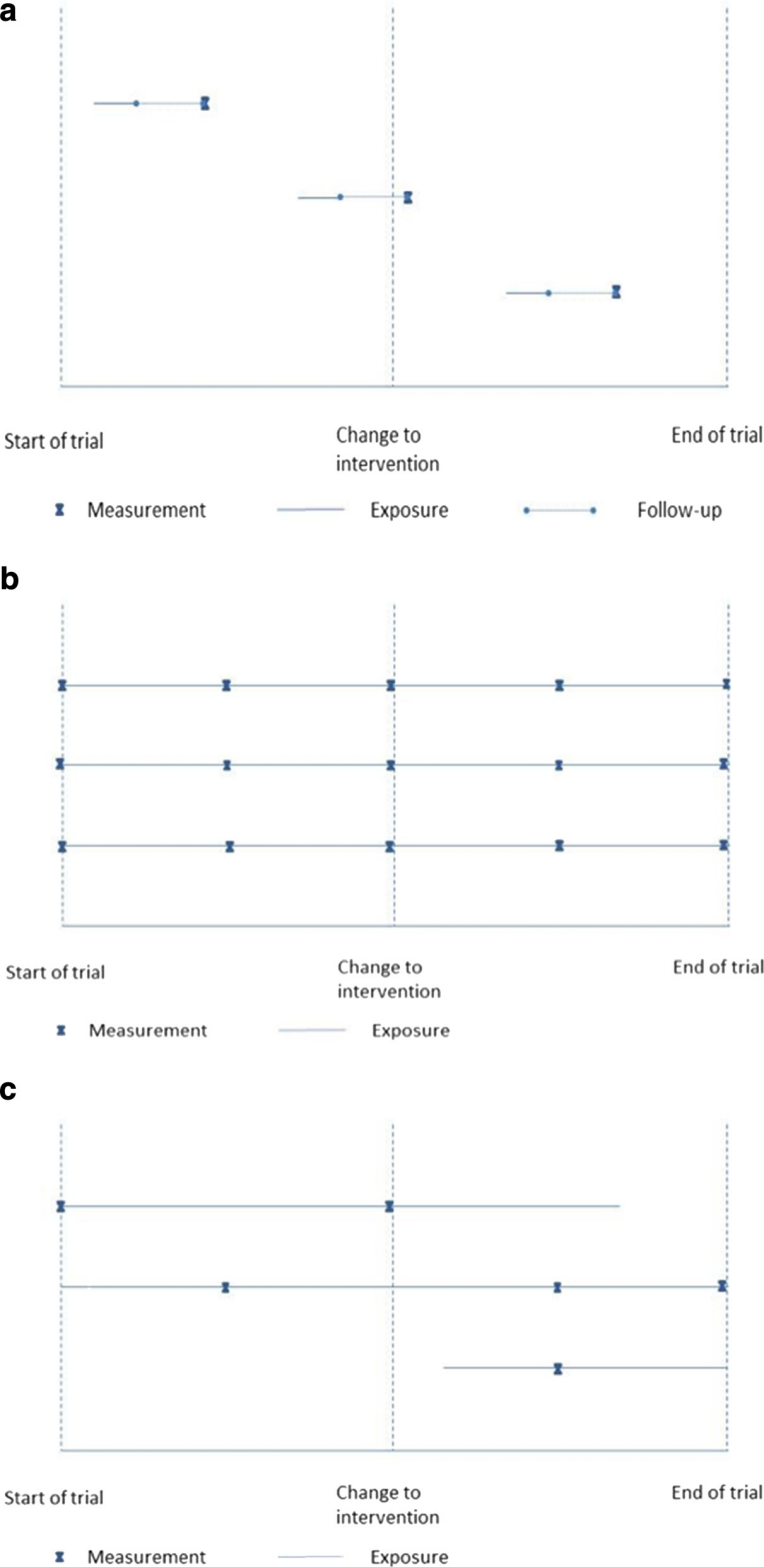
Diagrams to represent the exposure and timing of measurement for three illustrative participants in each of three main designs: **a** the continuous recruitment short exposure design; **b** the closed cohort design with five measurements per participant, **c** the open cohort design with one to three measurements per participant

Thirteen of the trials included in our review used this design. Few (or even no) individuals participate as the trial begins, but more become eligible and participate over time, and are then exposed for a short period. The outcome is often measured after a follow-up period. Figure 2a illustrates exposure and measurement for the design. The middle participant is exposed only to the control condition, although the outcome is recorded after the cluster has crossed over to the intervention condition. Single measurement, repeated measurements or time-to-event from the start of the individual’s exposure may be chosen to assess outcomes, depending on the research question.

##### Case study one

**Fig. 3:**
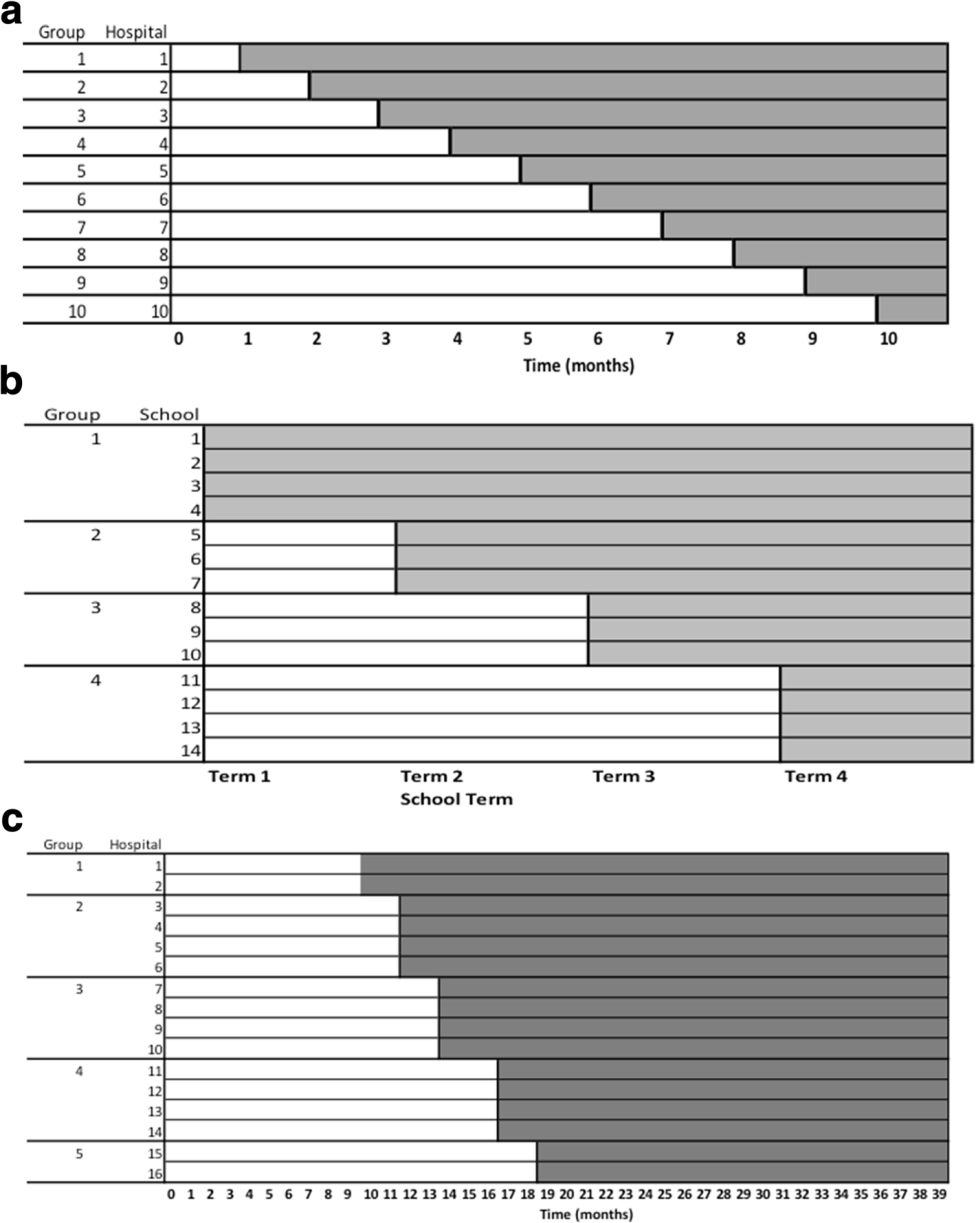
Diagrams to represent the rollout process in each of the three case studies, where shaded areas indicate intervention exposure and unshaded areas indicate control exposure: **a** case study one, **b** case study two, **c** case study three

Poldervaart *et al*. are conducting a trial to investigate the effect of introducing a policy promoting the use of a scoring system to guide clinical decisions for patients with acute chest pain on arrival at hospital emergency departments [9]. Ten hospitals were randomised, and one additional hospital implemented the intervention in each of 10 consecutive months (see Fig. 3a). Besides data collection during the rollout period, data were collected in the control condition from all hospitals for the first month of the trial, and from all hospitals once in the intervention condition in the final month. The primary outcome measured is the occurrence of a major adverse cardiac event within six weeks from presentation at hospital. The published protocol does not state whether patients may participate more than once in the trial, nor exactly what might happen to patients presenting just before a hospital changes to the intervention condition. However, as the exposure of the patient primarily relates to their management within the first few hours of arrival at hospital, it would seem that nearly all participants will be exposed to the control or intervention condition, and not both.

#### Closed cohort

Our review identified 11 trials with a closed cohort design. All participants are identified at the onset of the trial and participate from start till end, typically without any changing clusters. Repeated measurements are typically taken from the same individuals to assess change and its relation to exposure. Figure 2b illustrates a closed cohort design where individuals are measured repeatedly.

##### Case study two

Mhurchu *et al*. investigated the effect of providing free school breakfasts on pupils’ attendance [10]. Fourteen schools participated, and each school provided a closed cohort of pupils for one school year (Fig. 3b). After randomisation at the start of the school year, the intervention was rolled out to groups of three to four schools over four steps of length 2.5 months each (school terms). The primary outcome, school attendance, was assessed for each child and each term using a binary outcome indicating whether attendance was less than 95 % of that expected. No outcome data were collected when all of the clusters were in the control condition, but some were collected during the final term when all schools were in the intervention condition.

#### Open cohort

Our review identified 11 trials using an open cohort design. With this design, a substantial number of individuals are identified and participate from the start, but some may leave during the trial and others may become eligible and be exposed for some time. A minority of individuals may also change between trial clusters. Most participants will be exposed to both control and intervention conditions during the trial. Repeated measurements from the same individuals could be taken at times relating to their individual start of exposure, or researchers may choose to use a time-to-event outcome. However, these choices may be problematic in some trials due to individuals leaving the cohort over time, resulting in missing data. It is more common to use cross-sectional sampling of individuals at pre-specified times to provide data on the outcome of interest. Figure 2c illustrates exposure and measurement for an open cohort. In this case some individuals contribute more than one measurement. In other settings where clusters are large (such as cities), then only a very small proportion of participants are sampled for outcome measurement at each time, so individuals are measured once at most.

##### Case study three

Fuller *et al*. investigated the effect that providing feedback about hand hygiene to doctors and nurses would have on their compliance with protocol [11]. The study randomised 16 hospitals in groups of two to four to begin the intervention at one of five steps, with a median step length of two months and a total rollout period of nine months (Fig. 3c). Outcome data came from observations of staff compliance carried out every six weeks over the study period, and collected at the hospital ward level. The timing of these measurements does not seem to be linked to the trial steps or other aspects of the design. While it is not reported directly, we assume that there would be staff turnover during the trial as it is relatively long, and so we view this as an SWT with an open cohort design. Although the intervention is ‘delivered’ to staff, the outcome measurement is collected for a ward and pooled across the staff working the shift at that time. Data are collected over 39 months, including nine months before the rollout and 21 months after rollout has completed. Hence most of the period of data collection does not relate to the rollout period, which provides the most direct information concerning the effect of the intervention.

Our review also identified two trials with different designs to those described above. The first was conducted by Stern *et al*., and could be characterised as having continuous recruitment followed by long and varying periods of exposure [12]. The other trial by Williams *et al*. involved measurement only of patients first exposed shortly before a crossover point, and they are seemingly exposed to intervention or control, but not both [13].

In the introduction we mentioned that the design literature has focussed mainly on designs where measurements are obtained cross-sectionally at predefined discrete time points [2–5]. Now that we have outlined a range of SWT designs, we see that amongst recent trials the design literature mainly addresses two special cases: the open cohort design with only a very small proportion of participants sampled at each time point (so that participants are measured at most once), and the design of Williams *et al*. [13].

### How to describe exposure and measurement in a stepped wedge cluster randomised trial

We recommend that the design of an SWT should be described in terms of how individuals are exposed, including the start and duration of exposure, and whether some, all, or no individuals experience both the control and intervention, and how outcome measurements are obtained. Examples of each are given below.

#### Timing of start of exposure (T)

1. All individuals are exposed from the start.
2. Many individuals are exposed from the start, but some are first exposed later at various time points.
3. Groups of individuals are first exposed at one of a number of discrete time points.
4. No individuals are exposed at the start and they are first exposed in a continuous and gradual process.

#### Duration of exposure (D)

1. Through to close of trial.
2. Varying lengths across individuals.
3. Fixed length.

#### Measurement (M)

1. Repeated measurements from individuals, at fixed calendar times, possibly linked to the timing of the trial steps.
2. Repeated measurements from individuals, at times linked to the start of their individual exposure, for example at the start of exposure and at the end.
3. Cross-sectional measurement, at fixed calendar time(s), possibly linked to the timing of trial steps. This includes scenarios with repeated sampling at a low proportion from big clusters, so only a few individuals are sampled more than once.
4. Single measurement from each individual, at a certain time after the start of their exposure.
5. Time-to-event, where time begins at the start of exposure.
6. Number of events in an exposure period.

Referring back to our three main designs, we see that in this typology a closed cohort is typically T1/D1/M1. An open cohort with repeated cross-sectional sampling for outcome measurement is T2/D2/M3. The continuous recruitment short exposure period design is T4/D3 with either M4, M2, or M5. Given the timing and duration of exposure, there are often multiple choices of outcome measure types and data collection methods, but some would be inefficient or inappropriate. For example, cross-sectional measurement to assess change within a closed cohort (T1/D1/M3) is less sensitive than measuring the same individuals.

### Design choice one: number and length of steps

Trialists must choose the number of steps and the time between successive crossover points (or step length) with the total trial duration and sample size requirements in mind. Here we assume a complete design, meaning that data are collected from each cluster throughout the trial. In the next section we consider incomplete designs and analysis approaches, which can allow more flexible choice of the length and number of steps.

At least in the case of cross-sectional sampling and standard analysis methods, greater power is achieved with a higher number of steps [7], reaching a maximum when the size of each group is one cluster. Furthermore, in some trials it may be impossible to implement the intervention in more than one cluster at a time. However, conversely in other trials logistical constraints may prevent this, for example because each crossover point may induce training or other costs. In a closed cohort or open cohort SWT it is often desired to take measurements just before each crossover point, so a high number of steps may imply more measurement points and hence greater costs. In a closed cohort trial, in particular, this may also imply a high measurement burden on individual participants, and there may be little marginal gain in information from excessively increasing the number of measurements per individual.

Sometimes there is a lag period between when a cluster crosses over and when the intervention can affect the outcome in individuals. This may arise from a combination of implementation lag (delay until the intervention is fully implemented) and delay for the outcome to respond to the intervention. In open or closed cohort SWTs, the step length may be chosen so that the effect of the intervention in the group of clusters that most recently crossed over can be measured just before the next crossover point; therefore the length needs to be greater than the lag period. In a continuous recruitment short exposure SWT, the step length may be chosen to be large relative to the implementation lag period, so that in the group that most recently crossed over most outcome values from exposure before the next crossover point will be contributed by individuals exposed to the full intervention. Delay for the outcome to respond is not relevant for the short exposure continuous recruitment design, as individual follow-up may be long and its length is unrelated to the steps.

We recommend first investigating constraints on the number of steps and the minimum suitable step length given the lag period. If given these, a number of steps and step length can be found where the total trial duration is satisfactory and required sample size can be achieved, given the number of clusters considered, then the selections are finalised. This process can be iterative because the sample size required will depend on the number of steps [7]. Options described in the next section can be considered if there are no satisfactory selections of step length or duration.

In case study one it appears that the intervention has minimal implementation lag, and there are no restrictions on the number of steps, so the trial could be designed with the maximum number of steps, and step length set simply with the total trial duration and hence sample size in mind. In case study two, though a closed cohort, outcome data are obtained routinely so there are no restrictions on the number of steps arising from cost or measurement burden. The number of steps was, however, constrained to be no more than four by the preference to implement the intervention only at the start of school terms and conduct the trial in one school year, and step length was likewise constrained to be the length of the school term. With careful advance planning and publicity for the intervention, there need not be any implementation lag in case study two, and neither does it seem likely there would be any further delay for the intervention (providing breakfast) to affect the outcome (school attendance). In case study three there seems to have been no constraint on the number of steps, as data collection occurred at time points unrelated to steps. There was an implementation lag as staff training was required, but once training was received there seems no reason for a further delay for the intervention to affect the outcome of hand hygiene compliance. Had the implementation lag period been substantial (for example 10 weekly training sessions) and the more conventional approach of measurements before crossover points been taken, then it would have been natural to select step length to be slightly greater (for example more than 10 weeks) and then investigate the number of steps and its impact on sample size and power.

### Design choice two: incomplete or complete design

In the preceding section we have seen that a complete SWT may be of longer duration or fewer steps than wished, because a long step length is selected due to a lag period. Two approaches can be taken to shorten the step length, and possibly also total trial length, albeit potentially resulting in requiring more clusters in the trial. The first is to acknowledge the lag at the analysis stage [14], and is discussed by trialists in our companion paper [15]. Another solution at the design stage is to not collect data from clusters during the lag period, an incomplete design represented in Fig. 3 by Hemming *et al*. [3]. If measurements are taken before crossover points in a closed or open cohort SWT, then this approach allows the step length as selected for a complete SWT to be halved, as now we wish two step lengths to be greater than the lag period. This use of an incomplete design is worthy of consideration whenever there is a lag, that is, if the step duration for a complete SWT is longer than desired.

Incomplete designs have also been proposed to avoid measurement burden. For example in Fig. 2 of their paper, Hemming *et al*. describes a design where data are obtained from each cluster in the step before the crossover and for two steps afterwards [3]. Likewise within our review, the SWT conducted by Dreischulte *et al*. involves data collection from clusters only in certain periods before and after the crossover [16]. We feel unable to recommend these ‘sparse’ designs, with potentially few clusters providing data at each time point in the trial, until further confirmatory methodological work is conducted, but acknowledge their appeal.

### Design choice three: randomisation method

Two common problems faced by CRTs are imbalance in important characteristics across study arms despite randomising the clusters (particularly where the number of clusters randomised is small), and substantial reductions in power resulting from between-cluster variation. Two approaches taken to reduce both of these problems are matching and stratification [17]. Both approaches potentially reduce the between-cluster variation and improve balance and must be taken account of in the analysis. Stratification can also be used in the randomisation of the order of cluster rollout for an SWT. If clusters are divided into strata, the order of rollout can be randomised within each stratum. The numbers of clusters across the strata do not have to be equal. The Better Health Outcomes through Mentoring and Assessment (BHOMA) study is an SWT of a health systems strengthening intervention in Zambia, conducted in 42 clusters divided into three districts. There were seven clusters in district A, 14 clusters in district B, and 21 clusters in district C, so at each crossover point one cluster from district A, two from district B, and three from district C crossed over from the control to intervention [18]. As there were six clusters in each group, the stratification of the randomisation of clusters to groups assured balance of districts across the order of rollout. Analysis for the BHOMA study will then include district as a fixed effect. In this example, even though the numbers of clusters were unequal across strata, they were multiples of each other and at least one cluster from each stratum switched to intervention at each crossover point. This feature makes it feasible to include categorical time effects in the analysis that can be shared across strata, and hence simplifies the analysis. The equivalent of matching for an SWT would only be possible for SWTs with two steps. One trial protocol found in our review describes a more complex stratification, where some strata will have only two steps and the SWT conducted within strata may not overlap in time, resulting in a complex data structure and analysis [19].

Another approach to improving baseline balance in important variables for CRTs is restricted randomisation. In this approach, criteria for ‘reasonable’ baseline balance across arms are chosen and only randomisations that satisfy these criteria are ‘acceptable’. One of these acceptable randomisations is then chosen. Restricted randomisation can also be applied to an SWT, where the principle will be balance in the order of rollout, so that for example, the first half of clusters to cross over are not the most or least likely to have the outcome. Durovni *et al*. randomised 29 HIV clinics in an SWT with a primary outcome of tuberculosis incidence [20, 21]. Randomisation was restricted, such that ‘the sum of the covariate values weighted by the number of months in the intervention status must be within c_j_x100 % of that for control status’, where c_j_ determines how restrictive the criteria was, for six criteria: mean CD4 count, clinic size, average education, tuberculosis treatment levels, existence of a supervised tuberculosis therapy programme, and geography. For an SWT, in addition to determining how many acceptable allocations there are, one must also check whether any cluster is (almost) always allocated to the same point in the rollout order (that is, to one particular group) and whether any two clusters (almost) always appear in the same group (as then they are equivalent to just one cluster). A combination of stratified (for the variable most predictive of between-cluster variation) and then restricted randomisation (for other important variables) may be the best approach, and will be particularly important in SWTs with few clusters.

In a continuous recruitment short exposure design stratification and restricted randomisation could be based on characteristics of clusters, or of historical cluster summary values of outcomes or other characteristics of individuals within clusters. In a closed cohort design, besides this information, it may be possible to use cluster summary values of the characteristics of individuals who will participate in the trial if these are known before randomisation. In an open cohort design it may likewise be possible to use information from individuals who will participate at the start of the trial.

### Design choice four: collection of outcome data before or after the rollout period

In the design literature [2], the ‘classic’ SWT design includes one step length of data collection before rollout and one step length of data collection after rollout, as in case study one. There are two reasons why we do not, in general, recommend collecting (and then including in the primary analysis) more data from longer periods before or after rollout, particularly if this uses resources that could be used to collect more data during the rollout period. The first reason is that these data do not directly inform the estimation of the intervention effect unless strong assumptions are made concerning period effects before during and after the rollout period, which is inadvisable. Without such strong assumptions the gain in precision is modest, arising from information concerning variability between clusters. With increasing data before or after rollout the marginal gain in precision declines. The second reason is that including these data may introduce bias unless the model for these data over the data collection period is correctly specified, which is more difficult to do as the period becomes longer. Collecting data well after the rollout period may, however, be worthwhile if a secondary analysis of whether the intervention effect appears to be sustained is very important.

In case study three it seems most data included in analysis were collected before or after the rollout period. It would appear that focussing data collection on the rollout period, and perhaps extending the rollout period, would have provided a more informative trial. The collection of extensive data after the rollout period did, however, permit a per-protocol analysis. Conversely, in case study two it seems that since outcome data are routinely collected that data from immediately before the rollout period could have been compiled and included in analysis if the participants attended the same school before the year of the trial.

### Carry-over effects, bias, and individual exposure to one or both conditions

Carry-over effects are widely discussed in the literature on individually randomised crossover trials [22]. This design is commonly used with individuals with a chronic condition who are randomised to receiving a standard treatment for a certain period followed by a new treatment, or vice versa. Health is measured during each period to determine which treatment is better, and there may be a ‘wash-out’ period between the two treatments so that there will be no carry-over effect of the first treatment during the second period. The design is not normally recommended when carry-over effects are anticipated. Carry-over effects are always considered, and can arise if the new treatment has a permanent effect on the health of participants after a short period of administration.

Carry-over effects are the main reason why a crossover design is usually not recommended for CRTs that involve staff training interventions within health facilities. Staff cannot be ‘untrained’: in clusters randomised to receive the intervention first and the control condition afterwards, a carry-over effect from the training is likely to influence how patients will be managed, even when the intervention is formally withdrawn.

The issue of carry-over effects seems to have received very little attention in the literature on the SWT design, because the crossover is always from control to intervention, and so the obvious reasons to consider carry-over effects described earlier do not apply. Carry-over effects may also seem unlikely because in some SWT the control condition exists in the population before the trial, and so all participants have already been exposed to the control for so long that some additional exposure during the trial is unimportant. But carry-over effects of a somewhat different nature can nevertheless apply, and we feel these should always be considered, as in some cases the response to the intervention may be affected by a cluster’s duration in the control condition within the trial, or by the individual’s duration in the control condition if individuals experience both conditions within the trial.

Individual carry-over effects need to be considered in SWTs where many or all individuals experience both control and intervention conditions. In an open or closed cohort trial, carry-over effects can arise if the trial is conducted in a population where the outcome may not be stable. For example, in a trial of those diagnosed with a health concern, health may change during the control condition exposure, which could affect response to the intervention. For example, participants may become sicker through an extended period in the control condition, and hence be unable to respond fully to an improved treatment policy. This would lead to underestimation of the intervention effect. In our review we saw that such effects could potentially arise in trials of the management of diabetes [23, 24], amongst others. A carry-over effect can also arise in a continuous recruitment design if the exposure is of long duration: participants recruited during the control period for a cluster may switch care to the intervention policy part-way through. An example in our review compares methods to manage patients diagnosed with pressure ulcers during the trial [12].

Carry-over effects can also arise at the cluster level in trials comparing methods to detect a health condition and change its management. In such a scenario, the number of undetected cases remaining in clusters may decline over time. The types of undetected cases may also change, for example because cases that are more challenging to identify might remain undetected longer. The intervention and control conditions could therefore differ in how effectively cases are detected, and these changes over time will be influenced by the duration of the control condition. As the number and type of undetected cases will likely affect response to intervention there can be carry-over effects, most clearly in a closed cohort but also in an open cohort, unless individuals leave and join clusters at a high rate. This may be a concern in trials, such as one addressing detection and improved management of patients with multiple comorbidities and medications found in our review [25], or another trial involving identifying and treating depression in nursing homes [26]. This problem of changing participant distribution over time is most obvious for time-to-event outcomes such as death, and analysis of the intervention effect will be subject to survivor bias.

In case study two it seems unlikely that there will be a carry-over effect. Specifically the attendance of pupils in a term when the intervention is introduced (school breakfasts) is unlikely to be affected by whether a school had exposed pupils to the control condition (no breakfast) for one or two more terms more than in other schools. In case study three, carry-over effects are again unlikely as the control condition is a standard approach that staff will have experienced for a while before the trial, and the outcome is likely to remain stable.

Designs such as the continuous recruitment short exposure are more robust than the open or closed cohort designs because each individual experiences only one condition, so carry-over effects are less likely. Outcomes under the intervention condition are estimated only from individuals with no prior exposure to the control.

## Discussion

We have identified a wide range of SWT designs, classified in terms of how individuals experience control and/or intervention conditions, and how outcome measurements are obtained. These features are largely determined directly by the research question and setting. We have also offered guidance for the choice of key design features that are more directly under the control of researchers, such as the randomisation method, the number of steps, and the step length. This work describing the range of designs contrasts with the previous SWT design literature, which has generally focussed on designs where data are collected cross-sectionally at discrete time points. As shown in our review, these constitute only a minority of recently conducted trials [1].

As part of our work describing the range of SWT designs we have also outlined a set of features that researchers should report when describing their trial: (i) how individuals start their exposure; (ii) the duration of exposure; (iii) how individual exposure is influenced by the crossing over of the cluster to the intervention; (iv) how measurements are obtained; and (iv) whether the timing of measurement is linked to each individual’s exposure or to trial steps. Researchers may choose to use our suggested trial design names of closed cohort, open cohort, and continuous recruitment short exposure, but these do not replace providing a full description of exposure and measurement. Figures such as 2a-c may help to describe these aspects, particularly if the design is novel. We hope that describing these characteristics will become standard along with details of the allocation, for which a figure such as Fig. 3a-c is recommended, and the randomisation. This level of reporting would give additional important detail for some aspects beyond that recently suggested by others [8], and should be considered for future guidelines [6].

In our review, most conducted trials did not raise serious concerns for major carry-over effects, but it is unclear whether these were considered. We have described why the possibility of carry-over effects should be considered for SWTs in which individuals experience both control and intervention conditions. The continuous recruitment design is an attractive design since each participant experiences only one condition, and in many cases needs to provide only one outcome measurement. The closed cohort design, with repeated measurements on the same individual, may be problematic because individuals experience both conditions, but it can be a very powerful design. In many contexts, and in most trials in our review, it may be considered that since participants have been exposed to the control condition for a long time before the trial, they are likely to be stable in relation to the primary outcome. In this case it is unlikely that exposure to the control condition or duration on response to intervention will be an issue, so the standard analysis methods and interpretation will apply. Even if participants are stable at the start of the SWT, if the primary outcome is time-to-event (or rate of detection of a condition) then designs such as the closed cohort will always be susceptible to survivor bias. SWT designs where individuals experience both conditions may be a good choice, given constraints and the research question. In our opinion however, researchers should consider the possibility of carry-over effects and other bias *a priori*, and report these considerations when publishing the results of the trial.

Importantly, we have noted that in some SWTs outcome data are collected a long time before or after rollout, and then included in the primary analysis, such as in case study three. We think that this is generally inadvisable [14], and recommend collecting such data only if this does not reduce the data collected during the rollout period. Furthermore we suggest these data should be used only in an informal assessment of how the intervention changed the time trend of the outcome seen before rollout, or of whether the intervention effect is sustained beyond the rollout period.

Incomplete designs have been proposed in which data are not collected from all clusters at all times. These designs may be chosen to reduce cost and measurement burden or to reduce step length when there is a lag period between when a cluster crosses over and when the intervention can affect the outcome in individuals, so as to facilitate more steps in the trial or even a shorter overall trial length (whilst possibly increasing the number of clusters in the trial). Further work in this area might address other approaches to restricting the burden of measurement, for example collecting data from all clusters at all measurement times but varying the proportions of participants measured, so that measurement is unbalanced across clusters. One appealing option worthy of consideration could be to take measurements from a low proportion of participants from clusters in the exposure condition that is predominant at the time, that is, the proportion in intervention clusters would be high at the start, low at the end, and the same proportion as control clusters in the middle of rollout. This sampling option would increase the power from a ‘vertical’ analysis of the data [14].

We believe that a well-conducted SWT, in which participants experience only one condition and analysis appropriately takes account of period effects, provides strong evidence concerning the effectiveness of an intervention, and that this evidence will be far stronger than that from a non-randomised rollout. In our view, such a carefully designed and analysed SWT can in principle be as rigorous as a standard CRT, and deserves to be viewed as an experimental design rather than quasi-experimental. The quality of evidence provided by an SWT is a controversial topic, but we hope that this debate will become more nuanced, recognising that the SWT is a family of different designs which each have strengths and weaknesses.

## Conclusions

There is a wide range of stepped wedge trial designs, and key aspects such as the exposure of individuals and their measurement should be reported more clearly. Currently, simple randomisation is predominantly used, but researchers should consider the use of stratified and/or restricted randomisation. Trials should generally not commit resources to collect outcome data from individuals exposed a long time before or after the rollout period because these data contribute little to the primary analysis unless strong assumptions are made. Incomplete designs have been proposed and can allow a more flexible choice of the number of steps and step length. Though substantial carry-over effects are uncommon in stepped wedge trials, researchers should consider their possibility before conducting a trial in which individuals experience both control and intervention conditions, such as a closed or open cohort trial.

## Abbreviations

CRT: Cluster randomised controlled trial
SWT: Stepped wedge cluster randomized controlled trial
